# The complete chloroplast genome of *Prunus conradinae* (Rosaceae), a wild flowering cherry from China

**DOI:** 10.1080/23802359.2020.1768934

**Published:** 2020-05-22

**Authors:** Jiawen Yan, Jianhui Li, Wenfu Bai, Lin Yu, Dongling Nie, Zuheng Xiang, Sizheng Wu

**Affiliations:** a Institute of Economic Botany, Hunan Forest Botanical Garden, Changsha, China; b Forestry Bureau of Longshan County, Longshan, China

**Keywords:** *Prunus conradinae*, flowering cherries, chloroplast genome, phylogenomics

## Abstract

*Prunus conradinae* is a flowering cherry species with high ornamental value. In this study, the complete chloroplast (cp) genome of *P*. *conradinae* was obtained using a genome skimming approach. The cp genome was 158,019 bp long, with a large single-copy region of 85,910 bp and a small single-copy region of 19,247 bp separated by two inverted repeats of 26,431 bp. It encodes 130 genes, including 85 protein-coding genes, 37 tRNA genes, and eight ribosomal RNA genes. The phylogenetic analysis indicated that *P*. *conradinae* is closely related to the congeners *P*. *maximowiczii*, *P*. *takesimensis*, *P*. *speciosa*, *P*. *serrulata* var. *spontanea*, *P*. *discoidea*, and *P*. *matuurai*.

*Prunus conradinae* Koehne, also known as *Cerasus conradinae* (Koehne) Yü et Li, is a flowering cherry species with high ornamental value. It is mainly distributed in the Shanxi, Henan, Hubei, Hunan, Sichuan, Fujian, and Zhejiang province of China and usually grows in or on the edge of forests and valleys at 500–2100 m altitude, flowers in March (Wu and Raven 2003). A good knowledge of genome information and the phylogenetic position of *P. conradinae* would be essential to the formulation of efficient strategies for its conservation, exploitation, and utilization. To facilitate such purposes, the complete chloroplast (cp) genome of *P. conradinae* was assembled, and its phylogenetic status in *Prunus sensu lato* was analyzed.

The fresh leaves were collected from Yongshun county, Hunan province, China (28°47′28′′N, 110°16′36′′E). The voucher specimen (JIU04068) was deposited in the herbarium of Jishou University. Total genomic DNA was extracted by modified CTAB protocol (Doyle and Doyle 1987). The whole genome sequencing was performed on the Illumina Hiseq 2500 platform (Illumina, San Diego, CA). A total of 8.2 Gb clean reads were obtained, after removing low-quality reads by fastp software (Chen et al. 2018). Then, the clean reads were corrected by BFC tool (Li 2015). The processed data was assembled by GetOrganelle pipeline (https://github.com/Kinggerm/GetOrganelle), which exploits Bowtie (Langmead et al. 2009), BLAST (Camacho et al. 2009), and SPAdes (Bankevich et al. 2012) as dependencies. The cp genome was annotated by Dual Organellar GenoMe Annotator (DOGMA) (Wyman et al. 2004).

The cp genome of *P. conradinae* (GenBank accession number: MT374065) was 158,019 bp in length, consisting two inverted repeats (IR), a large single copy (LSC), and a small single copy (SSC), and the sequence lengths are 26,431 bp, 85,910 bp, and 19,247bp, respectively. The overall GC content of the cp genome is 36.7%, while the GC percentages in IR, LSC, and SSC are 42.5%, 34.6%, and 30.1%, respectively. The genome possesses 130 genes, including 85 protein-coding genes, 37 tRNA genes, and eight rRNA genes.

Phylogenetic analysis was conducted to confirm the phylogenetic position of newly sequenced *P. conradinae* amongst 22 representative *P*. *sensu lato* species and an outgroup taxa. We reconstructed a phylogeny employing the GTRGAMMA model and 1000 bootstrap (BS) replicates under the maximum-likelihood (ML) inference in RAxML (Stamatakis 2014). The ML tree (Figure 1) showed that all the species were divided into three clades (BS 100%), subg. *Cerasus*, subg. *Prunus*, and subg. *Padus*, which is consistent with previous results (Shi et al. 2013), and *P*. *conradinae* is closely related to the congeners *P*. *maximowiczii*, *P*. *takesimensis*, *P*. *speciosa*, *P*. *serrulata* var. *spontanea*, *P*. *discoidea,* and *P*. *matuurai*.

**Figure 1.**
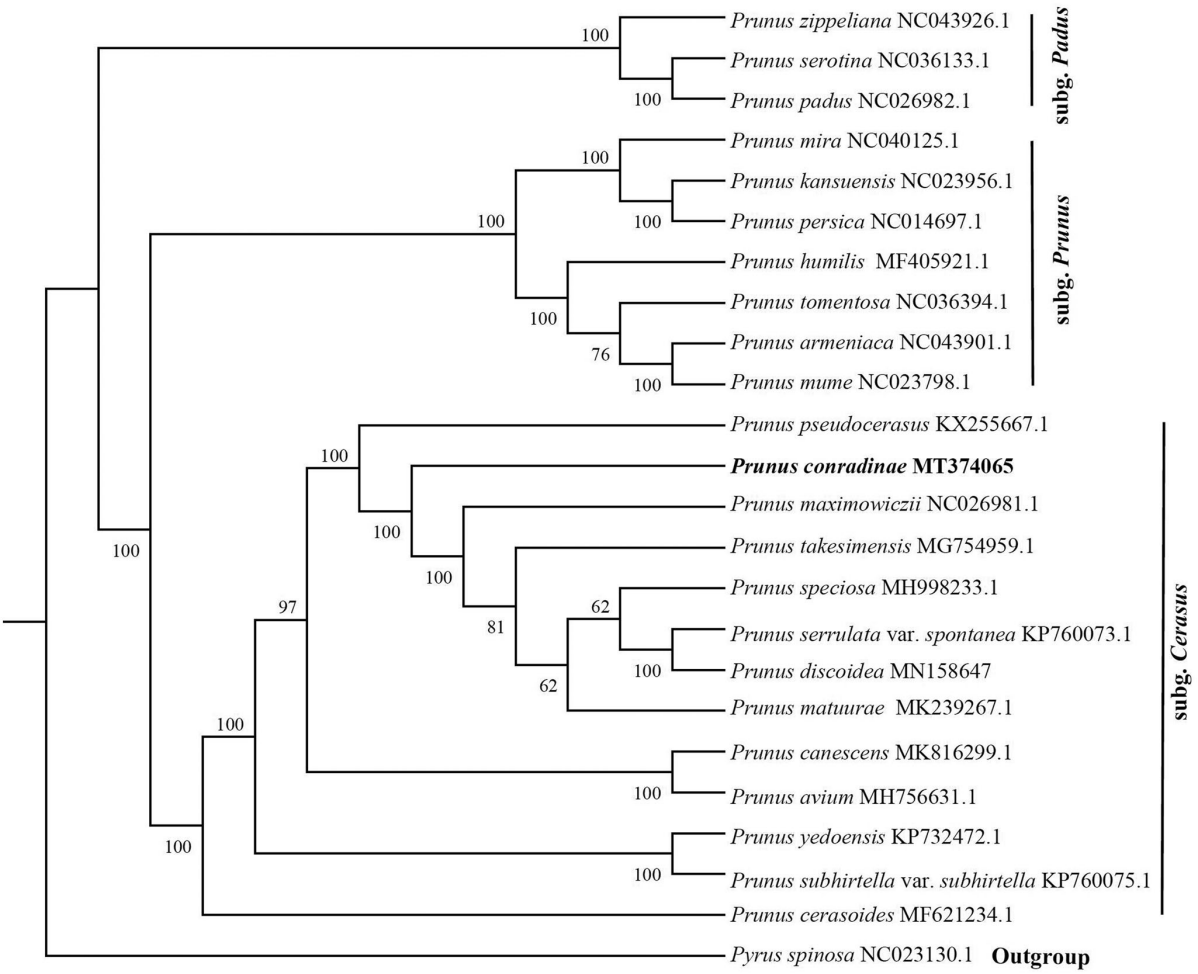
Maximum-likelihood phylogenetic tree for *Prunus conradinae* based on complete chloroplast genome of 22 taxa of *Prunus* *sensu lato*. *Pyrus spinosa* (Rosaceae) was used as outgroup. The support values are shown at the branches.

## Data Availability

The data that support the findings of this study are openly available in GenBank, National Center for Biotechnology Information (NCBI) at https://www.ncbi.nlm.nih.gov/genbank/, with accession number of MT374065.
